# Supplementary material for: Quantitative magnetic resonance imaging indicates brain tissue alterations in patients after liver transplantation
Source: PLoS One. 2019 Sep 25;14(9):e0222934. doi: 10.1371/journal.pone.0222934 (PMC6760889; doi:10.1371/journal.pone.0222934)
Supplement: S1 Table — (DOCX) [file pone.0222934.s001.docx]

**Supporting information**

**S1 Table. Results of the cognitive function testing with the RBANS.**

| n = 115 | CNI free  (group 1)  n=19 | CNI low  (group 2)  n=35 | CNI standard (group 3)  n=30 | control  (group 4)  n=31 | ***p*** | ***p2vs4****^1^* | ***p3vs4****^2^* |
| --- | --- | --- | --- | --- | --- | --- | --- |
| Immediate Memory median (IQ range) | 106 (20) | 103 (25) | 98.5 (30) | 109 (15) | *0.609* |  |  |
| Visuospatial/Constructional median (IQ range) | 96 (29) | 89 (31) | 96 (21) | 112 (34) | ***0.005*** | ***0.007*** | ***0.026*** |
| Language mean (±SD) | 101.37 (±12.8) | 98.49 (±12.81) | 99.97 (±12.78) | 105.19 (±9.63) | *0.143* |  |  |
| Attention mean (±SD) | 97.63 (±13.77) | 88.89 (±17.46) | 97.9 (±19.12) | 95.29 (±14.09) | *0.115* |  |  |
| Delayed Memory median (IQ range) | 101 (12) | 98 (11) | 99.5 (12) | 105 (13) | *0.085* |  |  |
| Sum of Index Score median (IQ range) | 508 (66) | 482 (±70) | 480 (64) | 519 (80) | ***0.021*** | ***0.019*** |  |
| Total Scale mean (±SD) | 100.79 (±12.23) | 92.6 (±13.3) | 96.73 (±14.67) | 103.48 (±13.72) | ***0.011*** | ***0.008*** |  |

^1^ Results of Tukey post-hoc testing of group 2 vs group 4. ^2^ Results of Tukey post-hoc testing of group 3 vs group 4. n, number; CNI, calcineurin inhibitors; RBANS, Repeatable Battery for the Assessment of Neuropsychological Status; IQ range, interquartile range; SD, standard deviation;
