# Supplementary material for: Quantitative magnetic resonance imaging indicates brain tissue alterations in patients after liver transplantation
Source: PLoS One. 2019 Sep 25;14(9):e0222934. doi: 10.1371/journal.pone.0222934 (PMC6760889; doi:10.1371/journal.pone.0222934)
Supplement: S2 Table — (DOCX) [file pone.0222934.s002.docx]

**S2 Table. Results of two-sided t-tests for parameter values measured in patients and controls^1^**

| Brain^2^ region | Patients | | | Controls | | | *p* |
| --- | --- | --- | --- | --- | --- | --- | --- |
|  | N | Mean | SD | N | Mean | SD |  |
| T2 (ms) | | | | | | | |
| BSd | 83 | 131.50 | 10.84 | 31 | 132.02 | 14.41 | 0.834 |
| BSv | 83 | 124.48 | 10.99 | 31 | 128.82 | 9.86 | 0.057 |
| Cb post | 83 | 118.83 | 5.28 | 31 | 118.26 | 5.38 | 0.611 |
| Cb sup | 85 | 120.30 | 6.08 | 31 | 121.18 | 6.37 | 0.499 |
| Cb inf | 85 | 115.80 | 6.17 | 31 | 113.67 | 4.24 | 0.079 |
| Cb ped. | 85 | 126.50 | 10.60 | 31 | 129.00 | 11.34 | 0.272 |
| CSO | 85 | 132.51 | 8.66 | 31 | 134.56 | 7.04 | 0.239 |
| fWM | 85 | 110.36 | 7.95 | 31 | 105.07 | 7.22 | 0.002 |
| GCC | 85 | 98.19 | 7.25 | 30 | 95.13 | 5.63 | 0.038 |
| GM occ. | 83 | 110.55 | 8.33 | 31 | 112.98 | 9.86 | 0.192 |
| Subcort. | 85 | 133.56 | 11.89 | 31 | 136.05 | 8.53 | 0.287 |
| Caud. nuc. | 84 | 98.38 | 7.18 | 31 | 96.87 | 5.69 | 0.294 |
| Pallidum | 84 | 74.48 | 7.35 | 29 | 72.63 | 4.56 | 0.117 |
| Putamen | 84 | 89.16 | 7.49 | 31 | 86.91 | 7.15 | 0.151 |
| pWM | 85 | 113.77 | 6.96 | 31 | 113.19 | 5.42 | 0.676 |
| SCC | 31 | 110.77 | 10.10 | 85 | 114.61 | 10.49 | 0.081 |
| Thalamus | 31 | 101.58 | 5.74 | 85 | 104.06 | 7.81 | 0.111 |
| T2* (ms) | | | | | | | |
| BSd | 85 | 31.12 | 5.55 | 31 | 31.89 | 6.55 | 0.533 |
| BSv | 75 | 22.25 | 8.13 | 29 | 20.53 | 6.38 | 0.310 |
| Cb post | 85 | 32.86 | 6.47 | 31 | 33.91 | 5.48 | 0.421 |
| Cb sup | 85 | 37.59 | 5.23 | 31 | 38.01 | 5.98 | 0.713 |
| Cb inf | 84 | 37.62 | 7.03 | 31 | 37.62 | 6.72 | 0.999 |
| Cb ped. | 84 | 34.50 | 6.36 | 29 | 32.95 | 6.20 | 0.257 |
| CSO | 85 | 44.65 | 4.93 | 31 | 44.52 | 5.95 | 0.909 |
| fWM | 85 | 44.26 | 4.90 | 31 | 40.83 | 4.91 | 0.001 |
| GCC | 85 | 40.37 | 5.77 | 30 | 37.31 | 4.31 | 0.009 |
| GM occ. | 85 | 36.88 | 4.80 | 30 | 37.04 | 3.86 | 0.872 |
| Subcort. | 84 | 40.06 | 6.07 | 31 | 40.00 | 5.77 | 0.961 |
| Caud. nuc. | 85 | 39.59 | 6.90 | 31 | 38.80 | 4.72 | 0.558 |
| Pallidum | 79 | 21.83 | 4.58 | 30 | 20.27 | 3.23 | 0.090 |
| Putamen | 84 | 30.87 | 5.53 | 31 | 29.74 | 4.81 | 0.318 |
| pWM | 85 | 40.22 | 5.79 | 31 | 39.02 | 4.59 | 0.301 |
| SCC | 84 | 34.99 | 4.69 | 31 | 34.50 | 6.45 | 0.705 |
| Thalamus | 84 | 38.03 | 5.18 | 31 | 36.40 | 3.83 | 0.113 |
| T2' (ms) | | | | | | | |
| BSd | 83 | 41.35 | 9.65 | 31 | 42.87 | 11.80 | 0.482 |
| BSv | 74 | 28.12 | 13.50 | 29 | 24.85 | 9.41 | 0.235 |
| Cb post | 83 | 46.39 | 12.68 | 31 | 48.34 | 11.47 | 0.457 |
| Cb sup | 85 | 55.41 | 11.46 | 31 | 56.12 | 12.74 | 0.776 |
| Cb inf | 84 | 57.07 | 15.32 | 31 | 57.59 | 15.77 | 0.874 |
| Cb ped. | 84 | 48.28 | 11.99 | 29 | 44.99 | 10.51 | 0.191 |
| CSO | 85 | 67.98 | 10.83 | 31 | 67.43 | 13.62 | 0.822 |
| fWM | 85 | 74.81 | 13.03 | 31 | 67.75 | 12.87 | 0.011 |
| GCC | 85 | 70.16 | 16.99 | 29 | 62.59 | 11.22 | 0.027 |
| GM occ. | 83 | 56.46 | 12.48 | 30 | 56.07 | 9.63 | 0.874 |
| Subcort. | 84 | 57.94 | 12.11 | 31 | 57.45 | 11.89 | 0.848 |
| Caud. nuc. | 84 | 67.81 | 19.42 | 31 | 65.50 | 12.31 | 0.538 |
| Pallidum | 78 | 31.66 | 9.58 | 28 | 28.51 | 6.31 | 0.109 |
| Putamen | 83 | 48.26 | 12.02 | 31 | 45.83 | 10.13 | 0.320 |
| pWM | 85 | 63.30 | 14.94 | 31 | 60.14 | 10.56 | 0.281 |
| SCC | 84 | 51.10 | 9.89 | 31 | 51.43 | 14.57 | 0.891 |
| Thalamus | 84 | 61.16 | 14.21 | 31 | 57.40 | 9.47 | 0.176 |
| ADC (x10^-6^ mm^2^s^-1^) | | | | | | | |
| BSd | 83 | 697.70 | 36.99 | 29 | 691.62 | 37.79 | 0.450 |
| BSv | 67 | 674.65 | 71.86 | 30 | 707.79 | 93.10 | 0.059 |
| Cb post | 85 | 661.46 | 50.59 | 31 | 670.82 | 62.30 | 0.410 |
| Cb sup | 83 | 669.39 | 43.38 | 31 | 664.30 | 44.67 | 0.581 |
| Cb inf | 85 | 631.44 | 33.51 | 31 | 620.94 | 27.76 | 0.122 |
| Cb ped. | 85 | 615.33 | 40.07 | 31 | 603.09 | 33.41 | 0.132 |
| CSO | 85 | 645.55 | 36.45 | 31 | 632.94 | 45.82 | 0.127 |
| fWM | 85 | 685.37 | 37.01 | 31 | 665.18 | 40.87 | 0.013 |
| GCC | 84 | 695.96 | 53.64 | 31 | 679.85 | 48.36 | 0.145 |
| GM occ. | 82 | 749.38 | 77.61 | 29 | 742.42 | 106.62 | 0.709 |
| Subcort. | 83 | 642.34 | 52.62 | 31 | 632.33 | 54.00 | 0.372 |
| Caud. nuc. | 80 | 644.37 | 43.31 | 31 | 635.40 | 47.24 | 0.342 |
| Pallidum | 82 | 753.97 | 90.33 | 31 | 770.98 | 135.69 | 0.442 |
| Putamen | 84 | 687.43 | 49.09 | 30 | 675.50 | 50.64 | 0.260 |
| pWM | 85 | 709.16 | 46.01 | 31 | 678.45 | 37.99 | 0.001 |
| SCC | 84 | 668.84 | 48.48 | 29 | 654.43 | 50.40 | 0.174 |
| Thalamus | 85 | 696.68 | 47.10 | 31 | 692.43 | 36.37 | 0.650 |
| FA | | | | | | | |
| BSd | 78 | 0.689 | 0.049 | 27 | 0.686 | 0.058 | 0.761 |
| BSv | 73 | 0.686 | 0.081 | 28 | 0.675 | 0.100 | 0.589 |
| Cb post | 55 | 0.232 | 0.044 | 22 | 0.221 | 0.053 | 0.367 |
| Cb sup | 41 | 0.219 | 0.035 | 23 | 0.223 | 0.042 | 0.691 |
| Cb inf | 52 | 0.254 | 0.042 | 20 | 0.240 | 0.043 | 0.229 |
| Cb ped. | 85 | 0.732 | 0.067 | 31 | 0.733 | 0.067 | 0.935 |
| CSO | 82 | 0.401 | 0.082 | 30 | 0.406 | 0.059 | 0.749 |
| fWM | 69 | 0.351 | 0.056 | 31 | 0.406 | 0.067 | 0.000 |
| GCC | 85 | 0.824 | 0.045 | 31 | 0.837 | 0.040 | 0.147 |
| GM occ. | 28 | 0.268 | 0.142 | 8 | 0.349 | 0.088 | 0.134 |
| Subcort. | 63 | 0.539 | 0.106 | 25 | 0.578 | 0.089 | 0.112 |
| Caud. nuc. | 43 | 0.195 | 0.032 | 19 | 0.192 | 0.026 | 0.764 |
| Pallidum | 30 | 0.328 | 0.071 | 11 | 0.373 | 0.104 | 0.121 |
| Putamen | 25 | 0.190 | 0.038 | 15 | 0.225 | 0.036 | 0.006 |
| pWM | 69 | 0.448 | 0.080 | 28 | 0.478 | 0.079 | 0.096 |
| SCC | 85 | 0.863 | 0.045 | 31 | 0.858 | 0.046 | 0.540 |
| Thalamus | 58 | 0.339 | 0.056 | 20 | 0.297 | 0.049 | 0.004 |

^1^ Results before corrections for multiple comparisons with false-discovery rate (FDR) method. Only those comparisons that have passed the corrections for multiple comparisons with FDR are considered significant in this manuscript. ^2^ Brain regions were the brain stem dorsal (BSd) and ventral (BSv), cerebellar posterior lobe (Cb post), superior (Cb sup) and inferior (Cb inf), cerebellar peduncle (Cb ped.), semiovale center (CSO), frontal white matter (fWM), genu of corpus callosum (GCC), occipital gray matter (GM occ.), subcortical white matter (Subcort.), caudate nucleus (Caud. nuc.), Pallidum, Putamen, parietal white matter (pWM), splenium of corpus callosum (SCC) and Thalamus. Note that several patients’ data were not considered for parameter FA analysis due to minor quality according to data quality criteria (SNR > 5). n, number; SD, standard deviation.
